# Ultrasound assessment of maternal risk factors for uterine scar dehiscence in repeat cesarean deliveries: a case-control study from Beirut clinic

**DOI:** 10.1097/MS9.0000000000005483

**Published:** 2026-08-06

**Authors:** Charlotte El Hajjar, Miralynn Awwad, Fayez Al Houssami Al Jobeily, Manal Dahrouj, Georges Yared, Kariman Ghazal

**Affiliations:** a Obstetrics and Gynecology Department, Lebanese American University, Beirut, Lebanon; b Obstetrics and Gynecology Department, Beirut Arabic University, Beirut, Lebanon; c Faculty of Medical Sciences, Paediatrics department, Lebanese University, Beirut, Lebanon; d Department of Obstetrics and Gynecology, Faculty of Medical Sciences, At Lebanese University, Beirut, Lebanon

**Keywords:** cesarean delivery, lower uterine segment thickness, obstetrics and gynecology, ultrasound assessment, uterine scar dehiscence

## Abstract

**Background:**

With rising cesarean delivery rates, uterine scar complications are becoming more common. Uterine scar dehiscence is often asymptomatic and is detected during repeat cesarean delivery, yet it carries risks of uterine rupture and adverse maternal and neonatal outcomes. Identifying maternal predictors and lower uterine segment (LUS) ultrasound measurements is essential in high-cesarean-rate settings, such as Lebanon.

**Objective:**

To evaluate maternal and obstetric factors and ultrasonographic LUS measurements associated with uterine scar dehiscence in women undergoing repeat cesarean delivery and to assess neonatal outcomes.

**Methods:**

This retrospective case-control study included 221 women who underwent a repeat cesarean delivery in 2023 at a private clinic in Lebanon. Cases (*n* = 117) had intraoperatively diagnosed uterine scar dehiscence, whereas controls (*n* = 104) had intact scars. Maternal factors, inter-delivery interval, bleeding during pregnancy, anemia, urinary tract infection, gestational age, and LUS thickness, measured by ultrasound, were analyzed. Multivariable logistic regression identified independent predictors.

**Results:**

LUS thickness <3 mm was strongly associated with uterine scar dehiscence (aOR, 13.49; 95% CI, 5.92–30.73; *P* = 0.001). Bleeding during pregnancy was also an independent predictor (aOR, 8.50; 95% CI, 3.47–20.85; *P* = 0.001), while a longer inter-delivery interval was protective (aOR, 0.80; 95% CI, 0.69–0.94; *P* = 0.006). Neonatal intensive care unit admission was higher among neonates born to mothers with dehiscence (16.2% vs. 5.8%; *P* = 0.014).

**Conclusion:**

Uterine scar dehiscence is associated with an LUS thickness <3 mm, bleeding during pregnancy, and shorter interdelivery intervals. Antenatal ultrasound assessment may improve risk stratification and outcomes in women with prior cesarean deliveries.

## Introduction

Cesarean delivery rates are increasing globally and now account for a substantial proportion of births. In Lebanon, cesarean deliveries account for 54.9% of total births, as reported by the Ministry of Public Health in 2024^[^1^]^. This high prevalence highlights the importance of understanding the long-term complications associated with uterine scars.

HIGHLIGHTS

- Uterine scar dehiscence was strongly associated with lower uterine segment thickness < 3 mm on antenatal ultrasound.
- Bleeding during the current pregnancy independently increased the risk of uterine scar dehiscence.
- A longer interdelivery interval was protective against uterine scar dehiscence.
- Neonates born to mothers with uterine scar dehiscence had higher rates of NICU admission.
- Antenatal ultrasound assessment may aid risk stratification in women with prior cesarean deliveries.

As cesarean rates increase, repeat cesareans are becoming more common, raising concerns about the integrity of scars and potential complications in subsequent pregnancies. Poor scar healing following a cesarean delivery may predispose women to scar dehiscence, uterine rupture, and abnormal placentation^[^2^]^.

Among these complications, uterine scar dehiscence is mostly identified at the time of a repeat cesarean^[^3^]^. It is frequently asymptomatic but can occasionally present with non-specific symptoms such as abdominal pain, scar tenderness, or vaginal bleeding^[^4^]^. It is a complication in which scar tissue remaining from a previous cesarean is disrupted and separates ^[^5^]^. When diagnosed before labor, dehiscence indicates an increased risk of intrapartum uterine rupture^[^3^]^. Uterine rupture involves direct communication between the amniotic and peritoneal cavities, leading to signs of intra-abdominal hemorrhage^[^3^]^. Although rare, uterine rupture can result in severe maternal and fetal morbidity and mortality^[^6^]^. Therefore, recognizing dehiscence is crucial for optimizing management and improving outcomes in women with prior cesarean deliveries.

Several maternal and obstetric factors may contribute to uterine scar dehiscence, including advanced maternal age^[^7^]^, a short interpregnancy interval^[^8^]^, multiple prior cesarean deliveries, anemia, and urinary tract infections (UTIs) in subsequent pregnancies. Understanding risk factors associated with dehiscence is important, especially in high-cesarean-rate settings, where women are more likely to undergo multiple repeat cesareans. This may not only increase maternal complications but also have adverse effects on neonatal outcomes, such as premature delivery and an increased rate of neonatal intensive care unit (NICU) admission.

Sonographic assessment of the uterine scar is clinically important for evaluating both scar thickness and integrity^[^9^]^. Measurement of lower uterine segment (LUS) thickness in women with a previous cesarean delivery has been shown to help identify patients at increased risk of uterine scar dehiscence or rupture. Studies have demonstrated a significant correlation between antenatal ultrasound findings and intraoperative assessment of the uterine scar, supporting the use of ultrasound as a reliable screening tool in women with a prior lower-segment cesarean section^[^10^]^.

Therefore, this retrospective case-control study aims to evaluate maternal and obstetric factors associated with cesarean scar dehiscence observed in a subsequent cesarean delivery, with antenatal ultrasound assessment of scar thickness. Women with documented dehiscence are compared with those with intact uterine scars to clarify these associations and optimize care for women with prior cesarean deliveries.

## Methodology

### Study design and population

This study was designed as a retrospective case-control study, conducted in accordance with the Strengthening the Reporting of Observational Studies in Epidemiology (STROBE) guidelines.

Medical records of 221 pregnant women who underwent repeat cesarean delivery at a tertiary referral center and presented to a private clinic during their pregnancy and follow-up period between January and December 2023 were included in the study.

Participants were classified into two groups based on intraoperative findings at the time of repeat cesarean delivery. The case group (*n* = 117) comprised patients with intraoperatively observed uterine scar dehiscence, while the control group (*n* = 104) comprised patients with an intact scar. Moreover, it is worth noting that both cases and controls were identified consecutively from the same population. They were also identified during the same study period, from January to December 2023. Indeed, all medical records of women undergoing repeat cesarean delivery were identified and reviewed. The group assignment was determined by a blinded obstetrician who was unaware of the antenatal LUS measurements at the time of the assessment. Non-dehiscence controls were selected from the same clinic population and study period to minimize selection bias. No matching was performed.

All patients’ details were de-identified before analysis to ensure anonymity. Participants provided verbal informed consent for the general use of their data for research purposes as part of routine clinical practice at their first presentation to the clinic. Written informed consent was waived by the Rafic Hariri University Hospital Institutional Review Board Committee due to the retrospective nature of the study.

The study was conducted in accordance with the ethical standards of Rafic Hariri University Hospital’s Institutional Review Board Committee.

All necessary ethical guidelines and standards were strictly adhered to, ensuring the integrity and ethical compliance of this study.

### Inclusion and exclusion criteria

Inclusion criteria were (a) pregnant women aged 18 years or older undergoing repeat cesarean delivery, (b) a low-transverse uterine incision at the previous cesarean delivery, and (c) availability of complete antenatal medical records.

Exclusion criteria were (a) multiple-gestation pregnancies, (b) pregnancies ending in fetal demise, and (c) previous non-cesarean uterine surgery.

### Exposures and outcomes

The primary outcome of this study was uterine scar dehiscence, defined as separation of a previous cesarean uterine scar without complete rupture, documented intraoperatively by the attending obstetrician. Primary exposures included maternal age, number of previous cesarean deliveries, inter-delivery interval, gestational age at the time of delivery, bleeding during the current pregnancy, maternal anemia, UTI during pregnancy, and ultrasound measurement of LUS thickness.

Maternal demographic variables included age, residence, educational level, and nationality. Educational level was categorized according to the level of formal education achieved by the participant. To elaborate, “low” included participants who had no formal education or attended only primary or intermediate school; “moderate” included secondary education or a technical diploma; “high” included university-level education or higher. Neonatal variables included NICU admission, which was considered a secondary outcome.

### Procedures

Routine antenatal care visits were conducted for all patients. During the first trimester, an ultrasound was performed to confirm an intrauterine pregnancy and to rule out a cesarean scar ectopic pregnancy. Subsequently, LUS thickness was measured transabdominally during third-trimester antenatal visits. The patient was in the supine position with a moderately full bladder. The transducer was placed in the suprapubic region in the sagittal plane.

For the present analysis, the LUS measurement obtained at the third-trimester visit closest to the scheduled delivery was used. The latter was adopted in order to standardize the gestational-age window and to correspond to the period during which the mode of delivery is typically decided.

The LUS was identified between the urinary bladder anteriorly and the amniotic cavity posteriorly. The thinnest portion of the anterior wall of the LUS at the level of the previous cesarean scar was measured. All measurements were performed by a single experienced operator using a Samsung R7 Expert ultrasound machine with a 2.5–3.5 MHz transducer. This approach eliminated interobserver variability and ensured consistent measurements across the study population.

LUS thickness was recorded and categorized as <3 mm or ≥3 mm. This categorization was adopted to ensure consistency and to minimize operator-dependent variability. In this study, transvaginal ultrasound was not used. Anemia, UTI, and bleeding during pregnancy were identified based on medical record documentation.****

### Study size

This study was designed as a retrospective observational study conducted at a single private clinic and a tertiary referral center, using all available patient records from January to December 2023. The sample size comprised all eligible patients who met the predefined criteria during the study period, totaling 221 participants. As this was a retrospective analysis, no a priori sample size calculation was performed.

### Statistical analysis

Statistical analyses were conducted using IBM SPSS Version 23.

Continuous variables were assessed for normality. Normally distributed continuous variables were presented as means with standard deviations and compared using the independent-samples *t*-test, while non-normally distributed continuous variables were presented as medians with interquartile ranges and compared using the Mann–Whitney *U* test. Categorical variables were presented as frequencies and percentages and compared using the chi-square test.

Multivariable logistic regression analysis was conducted to assess the association between potential risk factors and uterine scar dehiscence while adjusting for confounding factors. Variables included in the multivariable model were selected based on clinical relevance; these included maternal age, number of previous cesarean deliveries, interdelivery interval, gestational age at the time of delivery, bleeding during the current pregnancy, maternal anemia, UTI during pregnancy, and ultrasound measurement of LUS thickness. Results were reported as odds ratios (ORs) with 95% confidence intervals (CIs).

A *P*-value < 0.05 was considered statistically significant. Observations with missing data were excluded from the analysis.

## Results

### Participants

**Figure 1.**
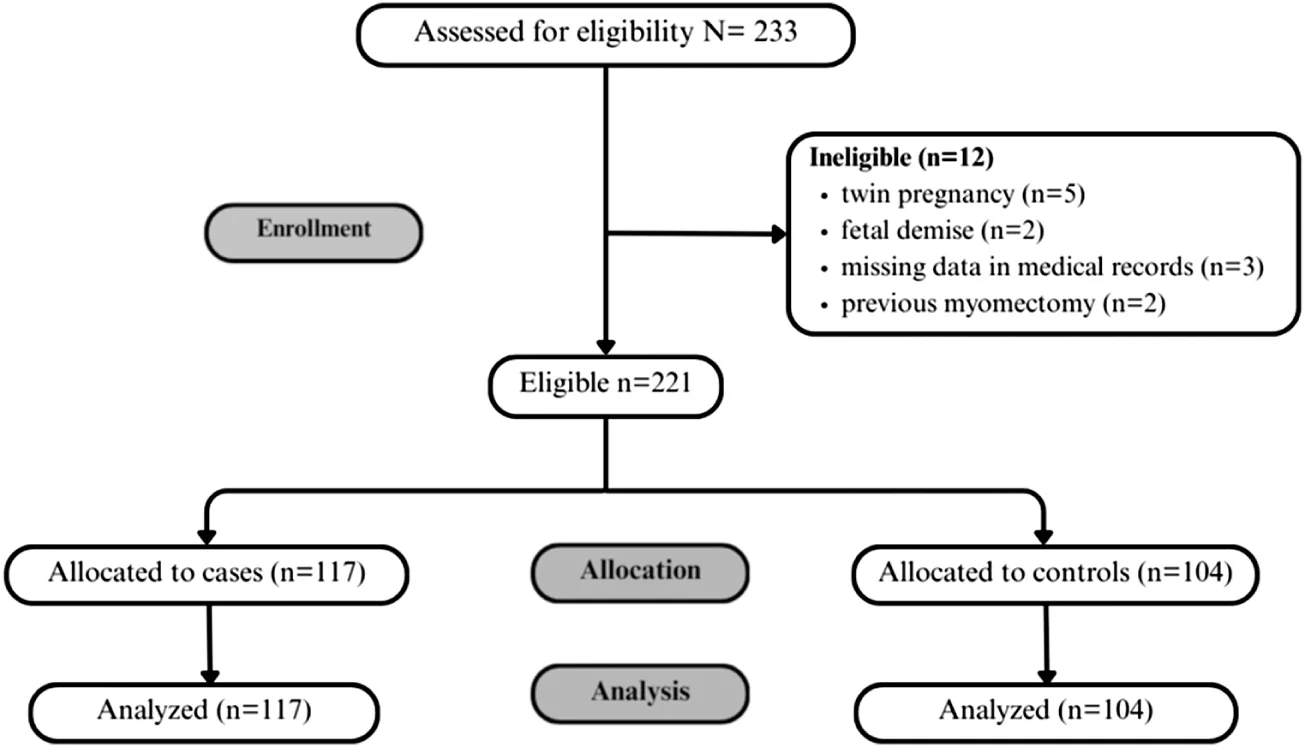
Participant flow diagram.

A total of 233 patients were assessed for eligibility. Of these, 12 were excluded based on predefined exclusion criteria. The remaining 221 patients met the inclusion criteria and were included in the analysis (Fig. 1). No follow-up was required, as this was a retrospective study.

### Descriptive data

Among the study population, which included 221 participants, the mean age was 30.48 years (SD = 4.45). Regarding the participants’ residence, 30.3% resided in urban areas, while the remaining 69.7% resided in rural areas. Regarding their educational level, 62% had a low educational level, 30.3% had a moderate educational level, and 7.7% had a high educational level. In terms of nationality, 39.3% were Lebanese, 49.7% were Syrian, and 11% had other nationalities (Table 1).

**Table 1:** Sociodemographic variables.

Sociodemographic variable	Patients (*n* = 221)
Age	
30.48 SD: ± 4.45	
Gender	
Female	221 (100%)
Nationality	
Lebanese	87 (39.3%)
Syrian	110 (49.7%)
Others	24 (11%)
Residency	
City	67 (30.3%)
Rural	154 (69.7%)
Educational level	
Low	137 (61.9%)
Moderate	67 (30.3%)
High	17 (7.8%)

No significant differences were observed between the cases and the controls for any demographic variable (p > 0.05). The mean age of participants with no dehiscence was 30.61 (SD ± 4.62), similar to that of participants with dehiscence, which was 30.35 (SD ± 4.31). The residential distribution was similar in both groups, with 28.84% and 31.62% of participants in the control and case groups, respectively, residing in the city, while the rest resided in rural areas. In terms of educational level, 67.3% of participants without dehiscence had a low educational level, 26.92% had a moderate level, and 5.76% had a high educational level. In contrast, 57.26% of the participants with dehiscence had a low educational level, 33.33% had a moderate level, and 9.4% had a high educational level. With respect to nationality, 33.65% of the participants without dehiscence were Lebanese, and 55.76% were Syrian. However, among participants with dehiscence, 44.44% were Lebanese, and an equal proportion (44.44%) were Syrian (Table 2).

**Table 2:** Sociodemographics of the Study Population.

Facto	Uterine scar dehiscence		*p-Value*
	Yes	No	
Age	Mean: 30.359	Median: 30.615	*0.670*
	SD: ± 4.31	SD: ± 4.62	
Educational Level
Low	67 (57.3%)	70 (67.3%)	*0.274*
Moderate	39 (33.3%)	28 (26.9%)
High	11 (9.4%)	6 (5.8%)
Nationality
Lebanese	52 (44.4%)	35 (33.7%)	*0.216*
Syrian	52 (44.4%)	58 (55.8%)
Others	13 (11.%)	11 (10.6%)
Residency
City	37 (31.6%)	30 (28.8%)	*0.663*
Rural	80 (68.4%)	74 (71.2%)

### Maternal and obstetric factors

A total of 221 women were included in the analysis and categorized according to the presence or absence of uterine scar dehiscence. The distribution of maternal and obstetric factors is presented in Table 3.

**Table 3:** Maternal and obstetric factors.

Factors	Uterine scar dehiscence	
	Yes	No
Number of previous Cesarean deliveries
One previous cesarean	49 (59%)	34 (41%)
Two Previous cesareans	34 (47.2%)	38 (52.8%)
Three or more previous cesareans	34 (51.5%)	32 (48.5%)
Ultrasound measurement of LUS scar thickness
Less than 3 mm	100 (70.9%)	41 (29.1%)
More than or equal to 3 mm	17 (21.3%)	63 (78.8%)
Anemia
Yes	108 (56.3%)	84 (43.8%)
No	9 (31%)	20 (69%)
Urinary tract infection
Yes	102 (55.7 %)	81 (44.3%)
No	15 (39.5%)	23 (60.5%)
Bleeding in current pregnancy
Yes	56 (77.8%)	16 (22.2%)
No	61 (40.9%)	88 (59.1%)
Interval since last cesarean delivery	Median: 3	Median: 4
	IQR: 2–4	IQR: 3–5.5
Gestational age at delivery	Median: 38	Median: 38
	IQR:37-38	IQR: 37.21-38

Among women with one previous cesarean delivery, 49 (59%) had uterine scar dehiscence, while 34 (41%) did not. Among women with two previous cesarean deliveries, 34 (47.2%) had uterine scar dehiscence, while 38 (52.8%) did not. Lastly, among women with three or more previous cesarean deliveries, the frequencies were 34 (51.5%) with uterine scar dehiscence and 32 (48.5%) without.

In women with uterine scar dehiscence, 100 (70.9%) had a LUS scar thickness <3 mm on ultrasound, while 17 (21.3%) had a thickness ≥3 mm. For those without dehiscence, only 41 (29.1%) had a thickness <3 mm. Diagnostic performance analysis of the <3 mm threshold yielded a sensitivity of 85.5%, a specificity of 60.6%, a positive predictive value (PPV) of 70.9%, and a negative predictive value (NPV) of 78.8%. The crude OR for LUS scar thickness <3 mm was OR = 9.0. Accordingly, women with a scar thickness <3 mm were approximately nine times more likely to experience dehiscence than those with a thickness ≥3 mm.

Anemia was present in 108 women (56.3%) with scar dehiscence and in 84 women (43.8%) without dehiscence. Additionally, UTIs were observed in 55.7% of women with dehiscence and in 44.3% of those without dehiscence.

The median interval since the last cesarean delivery was shorter among women with uterine scar dehiscence (median 3 years, IQR 2–4) than among those without dehiscence (median 4 years, IQR 3–5.5). Gestational age at delivery was similar between the two groups, with a median of 38 weeks in both groups.

Bleeding during the current pregnancy was more frequent among women with scar dehiscence, occurring in 56 women (77.8%) compared with 16 (22.2%) among those without dehiscence. The crude OR for bleeding during the current pregnancy was OR = 5.05. Accordingly, women with antepartum bleeding were approximately five times more likely to experience dehiscence.

### Multivariate analysis of factors associated with scar dehiscence

Of the factors studied for association with uterine scar dehiscence, bleeding during the current pregnancy (aOR 8.5, 95% CI 3.47–20.85, *P* < 0.001) and an LUS scar thickness < 3 mm on ultrasound (aOR 13.49, 95% CI: 5.92–30.73, *P* < 0.001) remained independently associated with uterine scar dehiscence after adjustment for potential confounders.

Additionally, a longer interval since the last cesarean delivery was independently associated with a lower risk of uterine scar dehiscence (aOR, 0.80; 95% CI, 0.69–0.94; *P* = 0.006). However, maternal age, anemia, UTI, gestational age at delivery, and the number of previous cesarean deliveries were not associated with uterine scar dehiscence (Table 4).

**Table 4:** Multivariate analysis of factors associated with uterine scar dehiscence.

Variable	Adjusted OR	95% CI	*p-Value*
Age	1.07	0.98-1.17	*0.110*
Anemia	1.69	0.51-5.58	*0.383*
UTI	0.99	0.35-2.75	*0.988*
Number of previous cesareans			
1 (reference)	–	–	*0.760*
2	0.78	0.33–1.83	*0.578*
≥3	1.07	0.42–2.07	*0.885*
Interval since last cesarean	0.80	0.68–0.93	***0.006***
Bleeding in current pregnancy	8.5	3.46–20.84	***<0.001***
LUS scar ultra-sound measurement	13.48	5.91-30.73	***<0.001***
Gestational age at delivery	1.07	0.74-1.55	*0.7*

Bold values indicate statistically significant results (*P* < 0.05).

The overall model was statistically significant in omnibus testing (*P* < 0.001) and demonstrated good calibration according to the Hosmer–Lemeshow goodness-of-fit test (*P* = 0.096).

### Neonatal outcome

Neonatal outcomes were assessed as a secondary outcome of the study. The neonates born to all study participants were included in the analysis (*N* = 221). Overall, 25 (11.3%) of the neonates required admission to the neonatal intensive care unit (NICU). Admission occurred more frequently among neonates born to mothers with intraoperatively observed dehiscence, 19 (16.2%), compared with those without dehiscence, 6 (5.8%). This difference was statistically significant, as indicated by a *P*-value of 0.014 using the Pearson χ^2^ test, indicating an association between maternal uterine scar dehiscence and NICU admission in neonates (Table 5). The crude OR for NICU admission was OR = 3.17. Accordingly, neonates born to mothers with scar dehiscence were approximately three times more likely to require intensive neonatal care.

**Table 5:** NICU admission.

	NICU admission		*p-Value*
	Yes	No	
Uterine
Yes	19 (16.2%)	98 (83.8%)	***0.014***
Scar
No	6 (5.8%)	98 (94.2%)
Dehiscence

Bold values indicate statistically significant results (*P* < 0.05).

## Discussion

Cesarean deliveries, especially repeat cesarean deliveries, are associated with an increased risk of uterine rupture, abnormal placental implantation, placental abruption, and uterine scar dehiscence in subsequent pregnancies^[^11^]^. These complications highlight the long-term implications of repeat cesarean deliveries for maternal and fetal health, underscoring the need to identify risk factors and implement preventive strategies in routine obstetric care to minimize adverse outcomes.

Full-thickness uterine injury resulting from prior cesarean delivery initiates a healing process that may be abnormal, leading to elastosis, tissue edema, and myofiber disarray^[^12^]^. This altered healing process results in extensive architectural remodeling of the LUS and, in subsequent pregnancies, is associated with uterine dehiscence^[^13^]^ characterized by reduced smooth muscle content, increased collagen deposition, and dysregulated growth factor expression, ultimately resulting in diminished scar tensile strength and compromised uterine integrity^[^14^]^.

Our study identified several important predictors of uterine scar dehiscence in women undergoing repeat cesarean delivery. Notably, a thinner LUS scar measuring <3 mm on ultrasound and bleeding during the current pregnancy were strongly associated with dehiscence, while a longer interdelivery interval appeared protective.

A key observation in this study was that a scar measuring <3 mm on ultrasound was associated with a higher risk of intraoperatively observed scar dehiscence (p < 0.001), corresponding to a 13-fold increase in the odds of dehiscence (aOR, 13.49; 95% CI, 5.92–30.73). These findings are consistent with those of Tazion et al. (2018)^[^9^]^, who observed that no participants with an ultrasound measurement >3 mm showed intraoperative dehiscence, whereas 83.3% of those with a measurement of 1–2 mm and 16.7% of those with a measurement of 2.1–3 mm had documented intraoperative dehiscence. The study by Tazion et al. (2018)^[^9^]^ demonstrated an association between ultrasound measurements and intraoperative findings (*P* = 0.001), which is consistent with the statistically significant association observed in our study.

Although our findings align directionally with prior work, the precise LUS cutoff reported in the literature varies. Indeed, it fluctuates from approximately 1.5–4.05 mm ^[^3^].^ This heterogeneity can be attributed to differences in the measurement approach (transabdominal vs. transvaginal). It can also be attributed to whether full LUS or myometrial thickness alone is measured, the gestational age at assessment, and the reference standard used. When the analyses are restricted to standardized methodology, the discriminative cutoff narrows to approximately 2.0–3.65 mm^[^3^]^. This range encompasses our 3 mm threshold. The magnitude of our adjusted OR (aOR, 13.49) is larger than that reported in several unselected prospective cohorts. This result is expected given our case-control design.

Bleeding during the current pregnancy was another strong, independent predictor of uterine scar dehiscence, with women experiencing bleeding during pregnancy showing an 8.5-fold increase in the odds of intraoperatively observed dehiscence (aOR 8.5, 95% CI 3.47–20.85, *P* < 0.001). Clinically, bleeding may indicate placental abnormalities or abnormal implantation near or over the scar, potentially compromising scar integrity. This association has not been well explored in the literature, highlighting a gap in current evidence.

A short interdelivery interval has been shown to be associated with an increased risk of dehiscence (aOR 0.802; 95% CI 0.68–0.93; *P* = 0.006). This aligns with findings from a study showing that longer interpregnancy intervals (>18 months) were associated with thicker LUS measurements (5.14 ± 0.42 mm)^[^15^]^. Evidence suggests that maternal education plays a key role in improving awareness of the spacing of pregnancies. In a study conducted by Yadav B. (2018), educating women significantly increased their understanding of the need for, and benefits of, adequate spacing between pregnancies^[^16^]^. Therefore, incorporating educational interventions and counseling during antenatal and postnatal care can help mothers make informed decisions about family planning and optimize outcomes for both mothers and neonates.

In our study, anemia did not show a statistically significant association with uterine scar dehiscence. However, this may reflect the limited sample size rather than a true absence of effect. A previous study demonstrated a higher incidence of scar dehiscence among anemic women, with increasing risk as anemia severity worsens^[^17^]^. This was linked to impaired collagen synthesis and delayed wound healing related to iron deficiency^[^17^]^. Therefore, anemia may still be a relevant factor in uterine scar integrity, warranting further investigation in larger studies.

In line with previous studies, our results did not demonstrate a statistically significant association between maternal age, number of previous deliveries, and gestational age at the time of delivery and uterine scar dehiscence^[^18^]^. UTIs during pregnancy were also not found to be associated with uterine scar dehiscence.

As a secondary outcome, our study demonstrated higher rates of NICU admission among neonates born to mothers with uterine scar dehiscence. This aligns with findings from other studies demonstrating increased rates of preterm delivery and NICU admission in cases of dehiscence^[^19^]^.

### Limitations

The study is a retrospective case-control study with a limited sample size determined by the available patient data. This design limits control over the accuracy and consistency of the data in medical records. Additionally, it was conducted at a single private clinic, which may limit the generalizability of the findings to other populations.

Furthermore, as an observational case-control study, this design is inherently susceptible to selection bias and residual confounding. Furthermore, while the multivariable model did adjust for the clinically relevant covariates available, several determinants of scar integrity were not recorded. These included maternal body mass index (BMI), previous uterine closure technique (single versus double layer), prior wound infection, and labor status at the previous cesarean.

In addition, ultrasounds were performed by a single operator. This maximized the consistency of measurements and removed interobserver variability as a potential source of error. Nonetheless, intraobserver reproducibility could not be formally quantified in this retrospective dataset.

Finally, multicenter, prospectively designed studies are needed to validate the <3 mm transabdominal threshold before applying it to other populations.

## Conclusion

This study demonstrates that uterine scar dehiscence after a repeat cesarean delivery is closely associated with several identifiable predictors, including a LUS thickness of less than 3 mm, a short interdelivery interval, and bleeding during pregnancy. These factors not only increase the risk of scar complications but also contribute to higher rates of NICU admission, highlighting the potential impact on both maternal and neonatal health. Recognizing and monitoring these risk factors during prenatal care can enable timely interventions, appropriate delivery planning, and improved maternal and neonatal outcomes. Ultimately, these findings emphasize the importance of individualized risk assessment and stratification among women with previous cesarean deliveries to reduce adverse outcomes.

## Data Availability

The datasets generated and/or analyzed during the current study are available from the corresponding author upon reasonable request.

## Research registration unique identifying number (UIN)

Not applicable. This is a retrospective observational case-control study.
